# A Novel Method for the Complex Tube System Reconstruction and Measurement

**DOI:** 10.3390/s21062207

**Published:** 2021-03-22

**Authors:** Xiang Guo, Xin Su, Yingtao Yuan, Tao Suo, Yan Liu

**Affiliations:** 1 School of Aeronautics, Northwestern Polytechnical University, Xi’an 710072, China; suxin.ds@mail.nwpu.edu.cn (X.S.); yuanyingtao@mail.nwpu.edu.cn (Y.Y.); suotao@nwpu.edu.cn (T.S.); yan_liu@mail.nwpu.edu.cn (Y.L.); 2 International Research Laboratory of Impact Dynamics and Its Engineering Application, Xi’an 710072, China; 3 Department of Science and Technology, Shenzhen University, Shenzhen 518061, China

**Keywords:** complex tube system, photogrammetry, convolutional neural network, epipolar geometry, 3D construction

## Abstract

Pipe structures are at the base of the entire industry. In the industry structure, heat and vibration are transmitted in each pipe. The minimum distance between each pipe is significant to the security. The assembly error and the deformation of the pipeline positions after multiple runs are significant problems. The reconstruction of the multi-pipe system is a critical technical difficulty in the complex tube system. In this paper, a new method for the multi-pipes structure inspection is presented. Images of the tube system are acquired from several positions. The photogrammetry technology calculates positions, and the necessary coordination of the structure is reconstructed. A convolution neural network is utilized to detect edges of tube-features. The new algorithm for tube identification and reconstruction is presented to extract the tube feature in the image and reconstruct the 3D parameters of all tubes in a multi-pipes structure. The accuracy of the algorithm is verified by simulation experiments. An actual engine of the aircraft is measured to verify the proposed method.

## 1. Introduction

Pipe structures are utilized in many fields, such as crude oil transmission, heating systems, the chemical industry, weapons, hydraulic pressure technology, and other applications. Some pipe structures like the pipes in aircraft engines need high accuracy in size and assembly. In the industry structure, heat and vibration are transmitted in each pipe. The minimum distance between each pipe is significant to the security.

**Figure 1:**
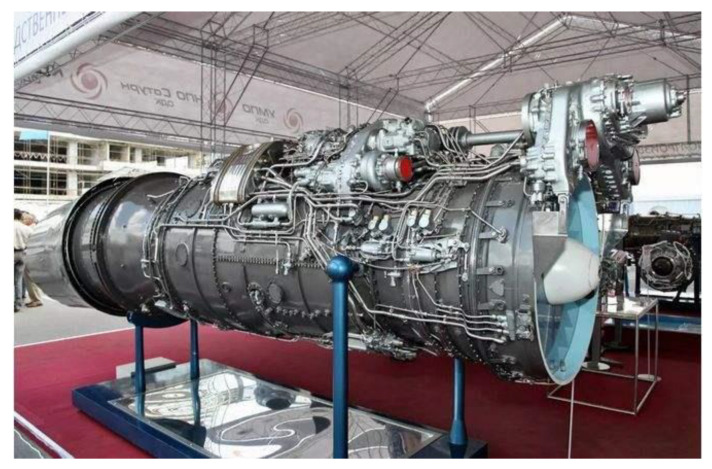
Complex tube system of the aircraft engine.

When pipe structures are manufactured and assembled, as shown in Figure 1, the assembly error and the deformation of the pipeline positions after multiple runs are significant problems for security. Several methods [1,2,3,4] are established to inspect the profile of the inner wall of a pipe. Meanwhile, inspection methods for pipe surfaces have been developed [5,6,7]. However, the presented methods are intended for the inspection of the single pipe. For the inspection of the multi-pipe system, there are still many problems to be resolved. The reconstruction of the multi-pipe system is a critical technical difficulty in the inspection of the multi-pipe system. For pipe structures, especially in the engine pipe structures, the size of structures differs. The fixed multi-cameras scheme is not universal and uneconomical to be utilized in the inspection of the multi-pipe system. In this paper, a scheme using a single camera based on Photogrammetry technology is proposed to inspect the multi-pipes structure.

Photogrammetry is defined as “the art, science, and technology of obtaining reliable information from noncontact imaging and other sensor systems about the Earth and its environment, and other physical objects and processes through recording, measuring, analyzing and representation” [8]. The definition of photogrammetry fits a variety of sensors to be employed. A widely accepted model for cameras is the pinhole model, which is described mathematically as a perspective projection to map 3D object space into two-dimensional image space. Depending on the distance between a camera and object, it can be distinguished as satellite photogrammetry, remote sensing, aerial photogrammetry, and close-range photogrammetry, where the distance is below a few hundred meters. There are many applications of close-range photogrammetry. The precise geometric information provided by close-range photogrammetry is frequently used in structural analyses.

In this paper, a new method for the multi-pipes structure inspection is presented. The images are acquired by one camera from different positions. The photogrammetry technology calculates positions, and the necessary coordination of the structure is reconstructed. A convolution neural network is utilized to detect edges of tube-features. The new algorithm for tube identification and reconstruction is presented to extract the tube feature in the image and reconstruct the 3D parameters of all tubes in a multi-pipes structure. The algorithm has been verified as accurate by a simulation experiment. An actual engine of the aircraft is measured to verify the proposed method.

## 2. Methods

**Figure 2:**
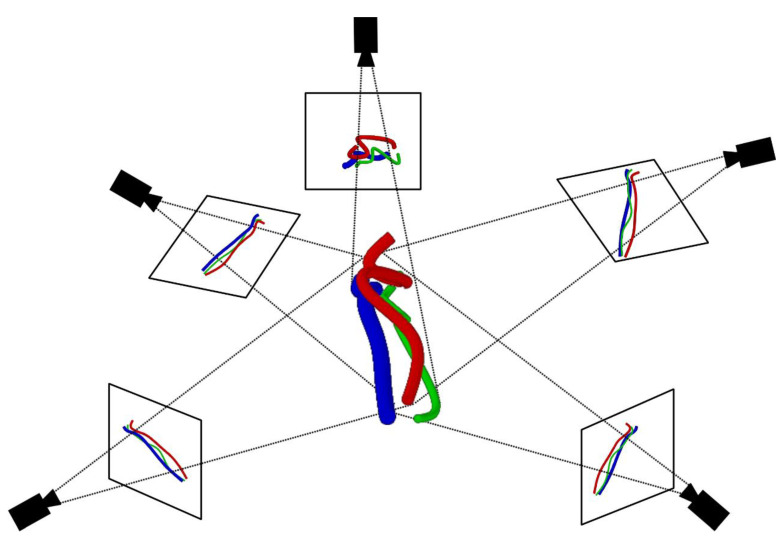
Complex tube system acquired by different camera locations.

For the detection of complex tube systems, the most critical problem is that there is overlap and background interference in two-dimensional images due to the pipeline complexity, as shown in Figure 2. How to obtain pipeline information in the image directly affects the accuracy of complex pipeline detection. Therefore, this paper presents a three-dimensional reconstruction method for the complex tube system.

**Figure 3:**
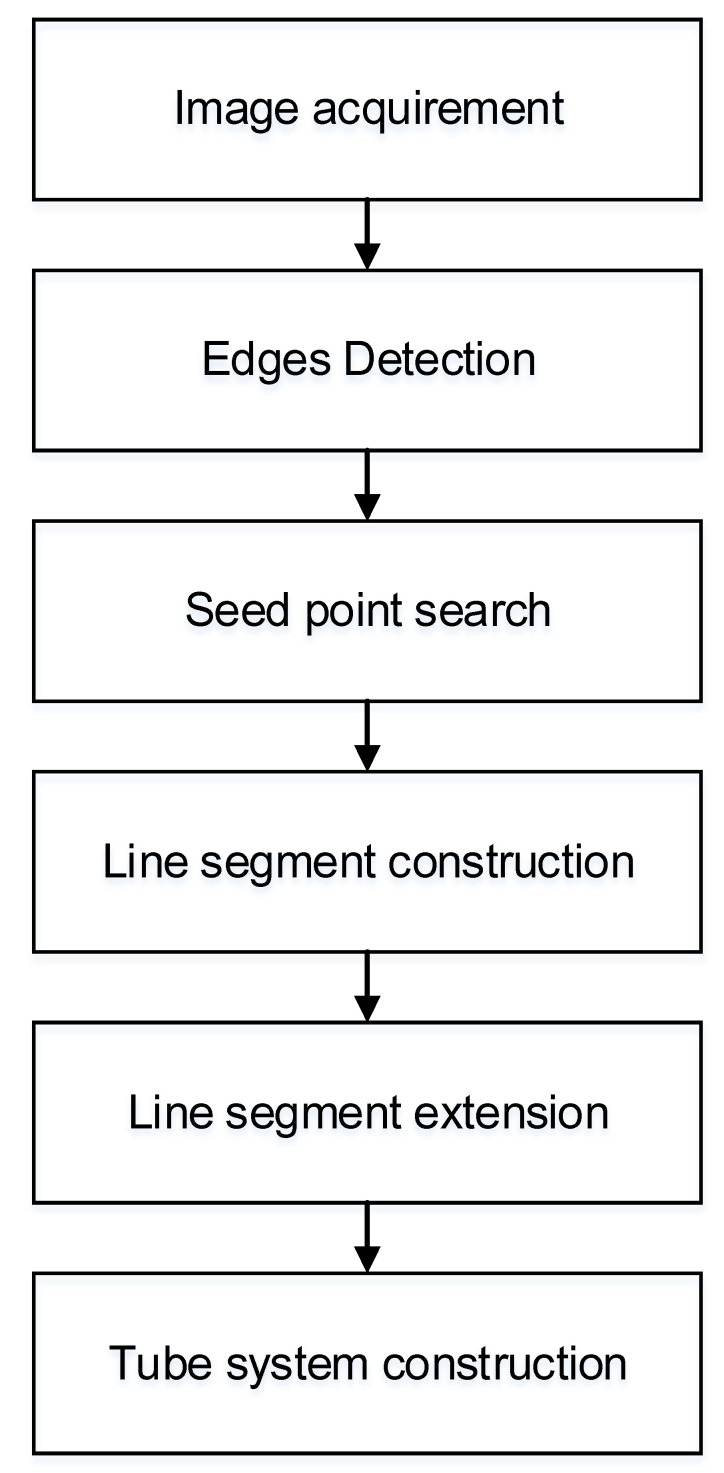
Scheme of tube reconstruction.

This paper mainly realizes the whole reconstruction of the complex tube system through five steps:

1. The coding mark points are placed around the tube system, the system is photographed from different angles and positions by photogrammetry technology, and the coding mark points placed around the system are combined to carry out photogrammetry reconstruction to obtain the camera 3D coordinates and optical axis angles of all shooting positions.
2. An edge-based neural network detects the edges of tubes.
3. A random edge is set as a seed point in tube detection.
4. The line segment is constructed based on the seed point and extended based on the spatial constraints judgment of the epipolar geometry.
5. Repeat steps 3 and 4 until all tubes are constructed. Finally, the tube system is constructed by the cylinder fitting. The 3D positions and radius of all tubes are calculated. The scheme of tube reconstruction is illustrated in Figure 3.

### 2.1. Camera Location Reconstruction

The photogrammetry technology can reconstruct the 3D coordinates of objects, based on a sequence of object images taken from different views. The mathematical model of photogrammetry technology is the well-known co-linearity equations [9]. By using the bundle adjustment [10], not only the 3D coordinates of objects but also the camera interior and exterior orientation can be obtained simultaneously.

In the measurement, coded and un-coded points are placed around the specimen. The digital camera acquires images of the specimen from different views. Image coordinates of coded and un-coded points are located by the image process, while IDs of the coded points are identified. According to image coordinates and IDs of the coded points, the 3D coordinates of all points are computed by the relative orientation, space resection, intersection, and bundle adjustment algorithms [9,10,11].

**Figure 4:**
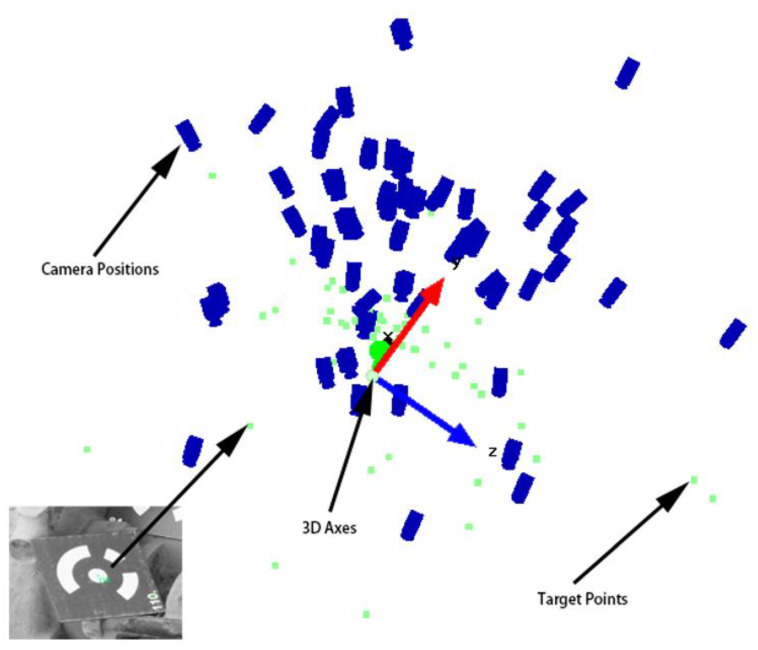
Positions of targets and cameras.

Figure 4 displays the camera coordinates and camera optical axis angles of all photographed positions during the reconstruction of a complex tube system. Green points are illustrated as the coordinates of coded targets. We acquired 76 images by the camera from different positions. In each image, more than five coded targets are acquired. Targets used in photogrammetry measurement are indicated as green points. Blue cameras indicate locations of camera acquirement. According to the coordinates of all cameras and optical axis angles, the 3D reconstruction of 2D images can be carried out, and the 3D reconstruction of the complex tube system will be carried out accordingly.

### 2.2. Edges Detection by the Convolutional Neural Network

In order to improve the accuracy of edge detection, the disturbance of the tube background should be eliminated. Unfortunately, none of the existing methods achieves the feature reliability and sharp boundary of desired objects. Traditional bottom-up methods mainly rely on priors or assumptions [12,13,14]. The deep convolutional neural network (CNN) has attracted wide attention for its superior performance [15,16,17,18,19]. Region-based CNN methods extract features of each region. However, existing region-based methods lack representing target information to model the relationship between similar tubes. Because of this, the detection results may fail to divide the edge of the tube submerged in complex tube-features.

**Figure 5:**
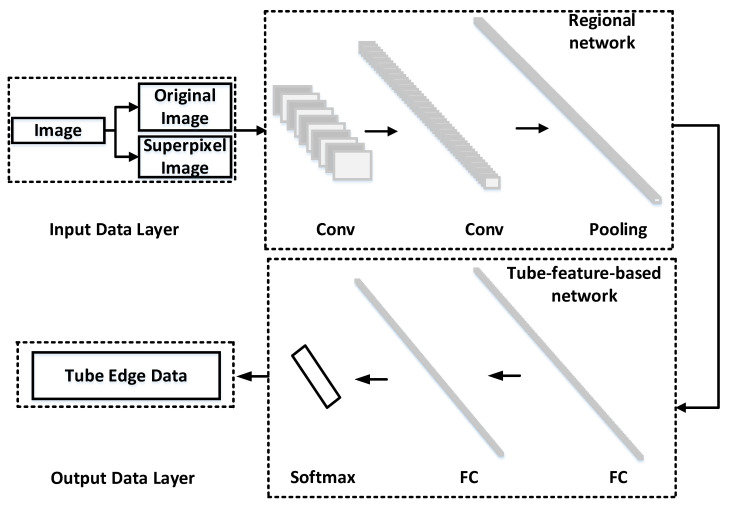
The convolutional neural network of edge detection.

A novel edge-preserving convolutional network for convoluted tube detection is presented in this paper to detect the edges of tubes. The proposed framework is mainly composed of a regional network and tube-feature-based network, as an illustration in Figure 5. The Fast R-CNN Framework inspires the regional network. The regional network achieves superior performances as a binary region classification task by the extraction of convolutional features in the entire image. The regional network segments the image into regions and predicts the edge threshold of each region of the image. The regional network obtains sharp boundaries. The tube-feature-based network achieves intensely reliable tube feature information of the image. In this paper, multiple convolutional layers are utilized to predict edges of different levels in the regional network, and edges of tubes are accurately located in the tube-feature-based network.

#### 2.2.1. Regional Network

A fast R-CNN is structured for edge detection. Firstly, an image is utilized as input. The image is segmented by superpixel and the original image. The superpixel segmentation algorithm is an improved image segmentation algorithm based on the SLIC and Niblack threshold method [20]. Secondly, each region segmented is used as input of a fast R-CNN framework similar to object detection tasks. Thirdly, a max-pooling layer converts the pooled feature to one value by using sub-windows. In this paper, the scale of the sub-windows is 11 × 11.

The operation of the convolutional layer is taken by:

(1) $$y_{i,j}^{\left(t\right)}=\tan h\left(\overset{m-1}{\underset{r=0}{\sum }}\overset{s-1}{\underset{k=0}{\sum }}\overset{s-1}{\underset{l=0}{\sum }}p_{i+k,j+l}^{\left(r\right)}\cdot w_{k,l}^{\left(r,t\right)}+b^{\left(t\right)}\right)$$

i=0,⋯,h−s; j=0,⋯,w−s; t=0,⋯,n−1,

where *p* is the position of the sub-windows, *s* is the side length of the square convolution kernels. *n* is the number of maps in the convolutional layer. m is the number of maps in the previous layer. *x* and *y* are the outputs of the previous and current layers. Parameters *h* and *w* are the height and width of the input region. *b* is a bias. For different output maps and different regions in the maps, the set of kernels and bias are different. Operator *C* is similar but has an additional abs operation after *tanh*.

A pooling layer is utilized to set the pooled features outside the region as 0:

(2) $$P_{j}=\{\begin{array}{cc} \underset{\left\{k|k\in SW_{j},M_{k}=i\right\}}{\max}F_{k} & i\in M\left(SW_{j}\right)\\ 0 & i\notin M\left(SW_{j}\right) \end{array}$$

where *i* is the index of the region. $SW_{j}$ is a certain sub-window. *M* is the region mask. *F* is the features before polling. *P* is the polled feature.

With the pooling layer, features of each region are extracted, and the edge information is preserved. Finally, the network generates an edge threshold of the entire image.

#### 2.2.2. Tube-Feature-Based Network

A tube-feature-based network is utilized in this paper to improve the reliability of edge detection. For the tube-feature-based network, two fully convolution layers are applied to increase the receptive field. Fully convolutional layers obtain the edge threshold.

The operation of the fully connected layer is taken by:

(3) $$y_{j}=\tanh\left(\overset{m-1}{\underset{i=0}{\sum }}p_{i}\cdot w_{i,j}+b_{j}\right),\;j=0,\cdots ,n-1,$$

where *n* and *m* are the numbers of neurons at the current layer and previous layer.

A softmax layer is utilized to obtain the final edge position:

(4) $$y=\mathrm{softmax}\left(\overset{3}{\underset{i=1}{\sum }}W_{f}^{i}\cdot h^{i}\left(\cdot \right)+b_{f}\right)$$

where $W_{f}^{i}$ and $b_{f}$ are the corresponding weights and bias in the softmax layer.

There are 4000 training images consisted of the training data. The training images consist of original images and the corresponding tube map. The input patch is taken as training patches with small translations and rotations. The threshold of edge detection is more than 85% of its neighborhood area is located inside the ground truth. Two loss functions are utilized in edge detection. One loss function is utilized to make the edge position *f* consistent with the tube map *M*.

(5) $$L_{1}=-average{\left(M_{i}\mathrm{lg}\left(f\right)+(1-M_{i})\mathrm{lg}\left(1-f\right)\right)}_{i=1\cdots 3}.$$

Another loss function is utilized to preserve the edge. For the segmented image, the edge threshold in the same region has a similar value. The standard deviation of the edge threshold is utilized to preserve the edge.

**Figure 6:**
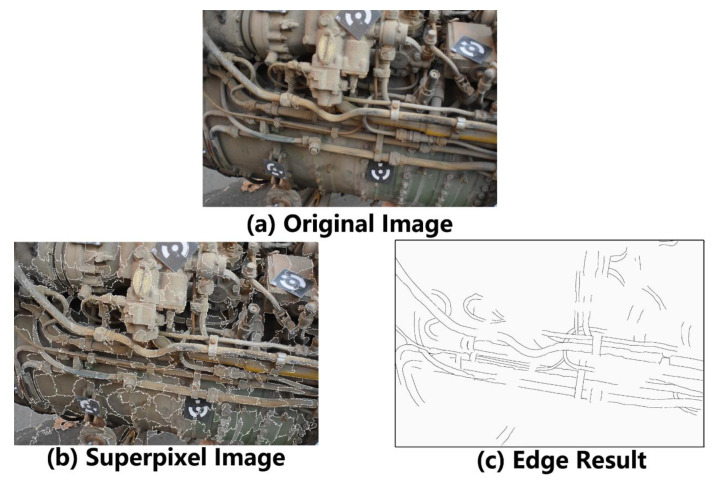
Input data and results of edge detection.

Figure 6 illustrates the input data and results of edge detection. The original image is utilized as input data of the convolutional neural network. An improved image segmentation algorithm obtains the superpixel image based on the SLIC and Niblack threshold method. The network detects the edges of the original image. Based on the edge detection results of each acquired image, the tube system will be reconstructed by seed point search and line segment construction.

### 2.3. Seed Point Search

Firstly, random seed points are placed in the image, and the seed points are randomly set up in the image through the intersection of the image grid to determine whether the edge information in the neighborhood of the seed points is a straight line segment of sufficient length and if the parallel length of the edge of the straight line segment and the threshold of straightness satisfy the condition. The resolution of the image decides the condition. The initial search is carried out as the seed point.

The straightness deviation is:

(6) $$Error_{Ln}=1-\left|\frac{n\sum_{1}^{n}(P_{x}^{i}\cdot P_{y}^{i})-\sum_{1}^{n}P_{x}^{i}\cdot \sum_{1}^{n}P_{y}^{i}}{\sqrt{\left[n\sum_{1}^{n}P_{x}^{i^{2}}-{\left(\sum_{1}^{n}P_{x}^{i}\right)}^{2}\right]\cdot \left[n\sum_{1}^{n}P_{y}^{i^{2}}-{\left(\sum_{1}^{n}P_{y}^{i}\right)}^{2}\right]}}\right|,$$

where $P_{x}^{i}$ and $\;P_{y}^{i}$ are the coordinates of the edge point. The linear fitting deviation should be less than the threshold value, and the linear fitting deviation threshold applied in this paper is 0.003.

### 2.4. Line Segment Construction

#### 2.4.1. Line Segment Construction Based on the Seed Point

**Figure 7:**
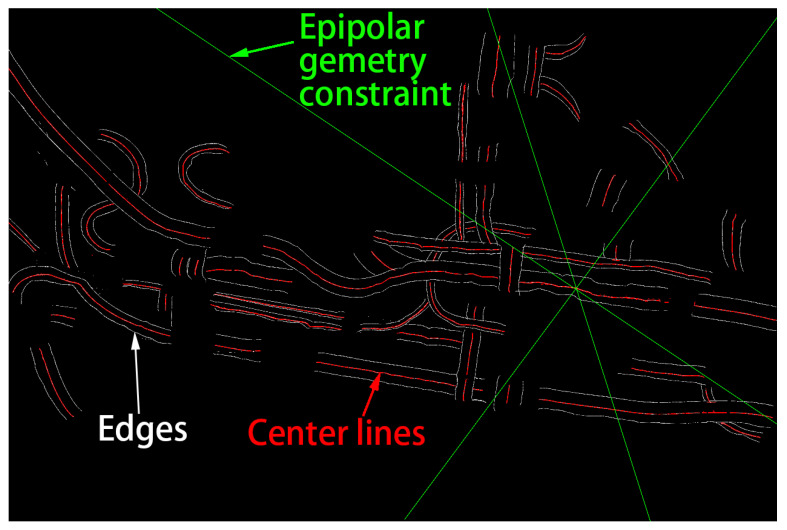
Epipolar geometry constrains in one image.

After the line segment of the seed point is calculated successfully, the center of the line segment of the seed point is used as the starting point of the 3D reconstruction. The edge data of the other images are searched according to the initial seed position and the epipolar geometry. The process is similar to the line segment calculation process in the line segment search, when there are more than three straight segments, using the epipolar geometry constraint (as shown in Figure 7) to confirm the correspondence between each segment. If there are multiple corresponding possible cases, the image constraint is increased until the individual matching results can be matched, if the individual matching results can not be obtained, then set the current seed point invalid and re-calculate another seed point.

When the seed point is determined, the straight section of the seed point is reconstructed in 3D. After reconstruction, the 3D coordinates are projected to each image. The 2D edges and center point coordinates in each image are recorded, and the 2D edges are marked as calculated states. This segment is marked as the initial line segment based on the seed point.

#### 2.4.2. Line Segment Extension

After the initial line segment is obtained, the segment is extended to obtain tube data in a complex tube system.

First, one side of the line segment is randomly selected as the extension direction, and the search space step is used as the reference to extend in 3D space. The coordinates of the extended point are projected to each image. During the bending segment, reduce the search spacing step size and fit the bending segment curve to identify and reconstruct the bending segment. During the bending segment, reduce the search spacing step size and fit the curve of the bending segment to identify and reconstruct the bending segment. When the corresponding 2D edge data cannot be matched after the extension, the one-direction search ends, and the search match in the other direction of the seed point is carried out. Repeat these steps for seed point calculations for other tubes until all bends are searched.

### 2.5. Tube System Construction

The main problem in the reconstruction of a complex tube system is to solve the overlapping of pipelines. Based on the photogrammetry stations information, this paper takes photos of the tube system from many angles by using multiple stations. The accurate extraction of the overlapping part of pipelines is realized. The reconstruction of pipelines is based on the constraint of the epipolar geometry of the multi-stations. When all the bend axes are calculated, the straight line sections and the bending sections are judged, the whole three-dimensional cylindrical fitting is carried out after the line section is fused, and the bending section is fitted by using a short step length to obtain the more accurate complex bend reconstruction results.

**Figure 8:**
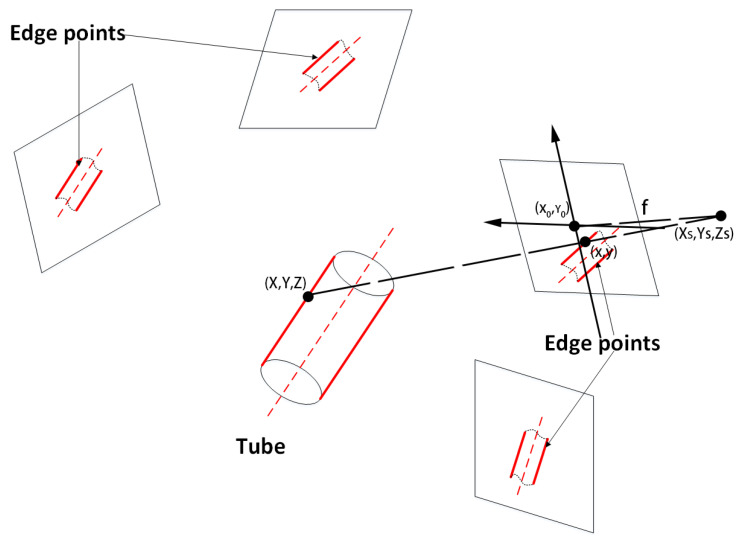
Cylinder fitting.

In Figure 8, the pose of the small cylinders should be optimized to match the cylindrical surface with edge points. The small cylinder in the world coordinate system needs to be projected onto every image repeatedly using the transformation between the world coordinate system and the camera coordinate system. The transformation function [21] is:

(7) $$x-x_{0}+\mathrm{dx}=-f\frac{a_{1}\left(X-X_{s}\right)+b_{1}\left(Y-Y_{s}\right)+c_{1}\left(Z-Z_{s}\right)}{a_{3}\left(X-X_{s}\right)+b_{3}\left(Y-Y_{s}\right)+c_{3}\left(Z-Z_{s}\right)}$$

(8) $$y-y_{0}+\mathrm{dy}=-f\frac{a_{2}\left(X-X_{s}\right)+b_{2}\left(Y-Y_{s}\right)+c_{2}\left(Z-Z_{s}\right)}{a_{3}\left(X-X_{s}\right)+b_{3}\left(Y-Y_{s}\right)+c_{3}\left(Z-Z_{s}\right)},$$

where (X,Y,Z) is the world coordinate of the object point, $\left(X_{s},Y_{s},Z_{s}\right)$ is the coordinate of the projection center, (x,y) is the coordinate of the image point, $\left(x_{0},y_{0}\right)$ is the coordinate of the image plane center, *f* is the length of the lens, *dx* and *dy* are the magnitudes of the lens distortion and matrix $\left[\begin{array}{ccc} a_{1} & b_{1} & c_{1}\\ a_{2} & b_{2} & c_{2}\\ a_{3} & b_{3} & c_{3} \end{array}\right]$ is the transformation matrix from the world coordinate to the camera coordinate.

The radius of the small cylinder is equal to the radius of tubes. The center line is used to represent the small cylinder. A projection center, optical center, and an edge point form a line $L_{j}$. Ideally, the distance between the center line and $L_{j}$ is equal to the radius. So, the center line can be determined by minimizing the equation:

(9) $$f=\min\overset{n}{\underset{i=1}{\sum }}\overset{m}{\underset{j=1}{\sum }}\left(d_{ij}-R\right),$$

where *i* is the number of cameras, j is the number of $L_{j}$ for a camera, $d_{ij}$ is the distance between the center line and $L_{j}$ in camera *i*, and *f* is the matching error used for evaluating the fit. The Levenberg–Marquardt algorithm [22] was utilized to solve it.

## 3. Experiment

### 3.1. Effectiveness Validation

A canny method [23] was utilized to obtain the edge data of images. The reconstruction result by the canny method was compared with the result constructed by the proposed method.

**Figure 9:**
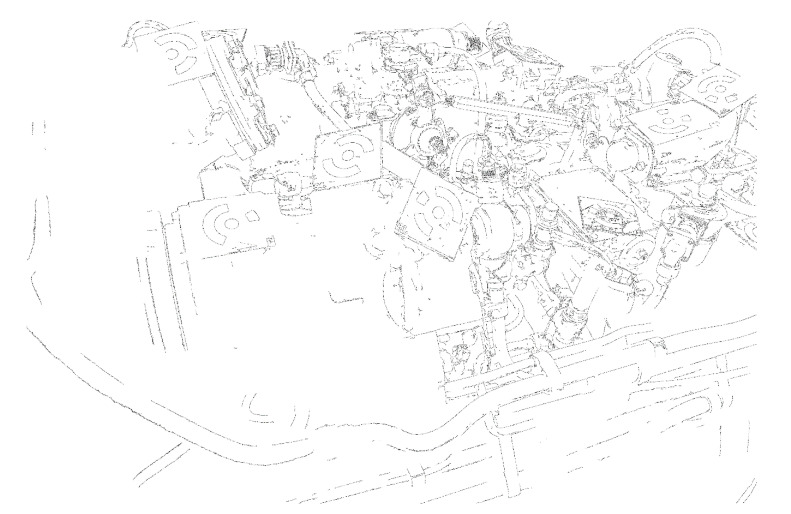
Edge result of the canny method.

**Figure 10:**
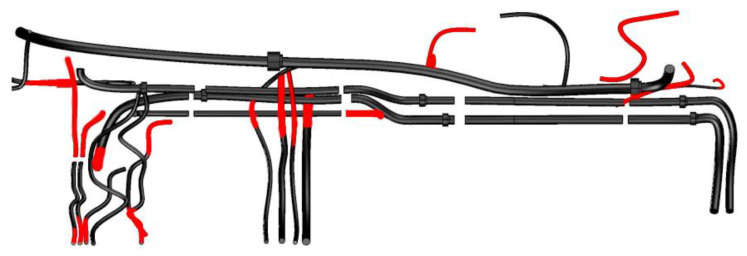
Comparison of the proposed method and canny method.

The edge detection result of the canny method is illustrated in Figure 9. Because of the complex background interference, the canny method cannot obtain stable and effective results of tube edges. Reconstruct the complex tube system by the result of the canny method, and compare the canny reconstruction result to the proposed method result Figure 10 illustrates the comparison of the proposed method and the canny method. Because of the loss of tube edges detected by the canny method, some pipelines were not reconstructed by the canny method. The black tube segments were similar, and the red segments were the canny method’s un-constructed tube segments. Compared with the canny method, the proposed method can obtain more stable and more effective results of the complex tube system.

**Figure 11:**
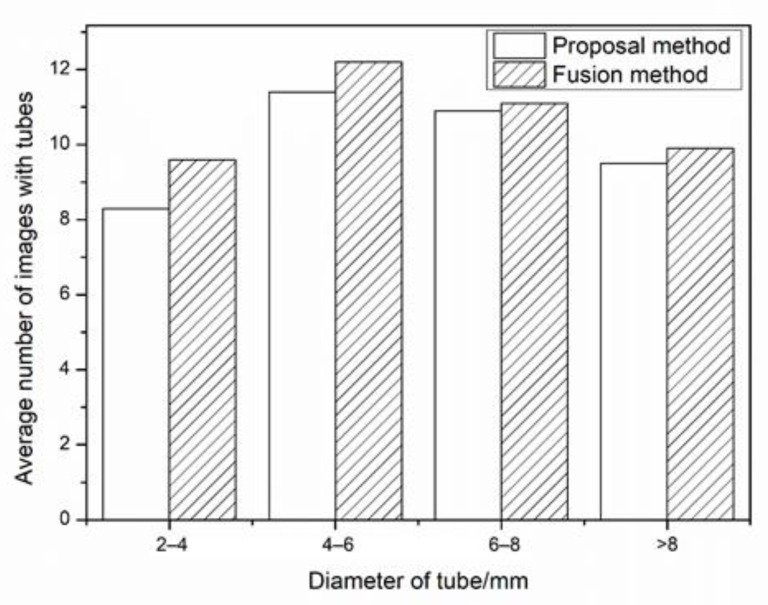
The average number of images with different tubes.

**Figure 12:**
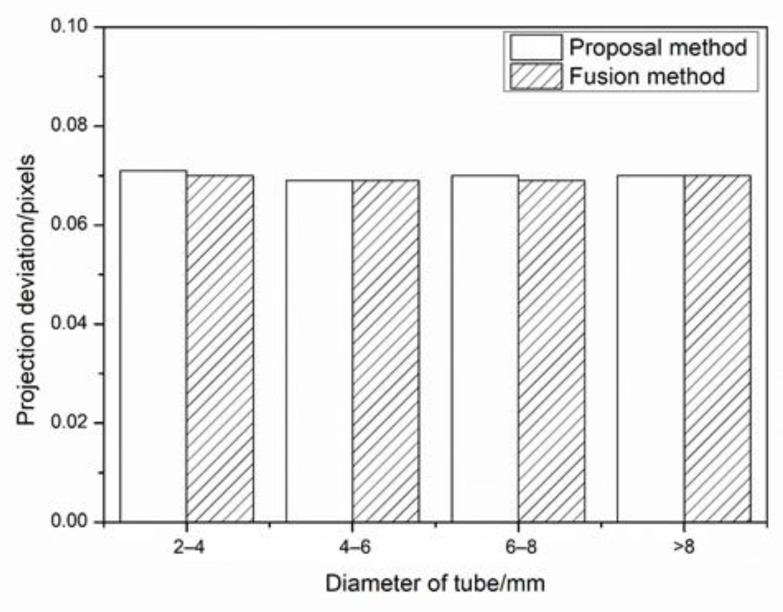
Projection deviation of the reconstruction results.

Compared with other CNN methods [20,24], a fusion method similar to the RexNet presented in [24], is utilized to calculate the edge of tubes. The average numbers of images with different tubes are illustrated in Figure 11. Because of multi-scale spatial detection layers, more edges of tubes are detected by the fusion method. Projecting the reconstructed tube coordinates to images, and calculate the deviation of edges of tubes. Figure 12 illustrates the projection deviation of the proposed method and fusion method. In the proposal method, more than seven images of each tube are obtained. The comparison of reconstruction results is similar in the projection deviation. Compared with the fusion method, there are four different scale layers and one fusion layer in the fusion method. For the computational complexity, the proposed method is more efficient than the fusion method. The proposed method has enough accuracy for the engineering application.

### 3.2. Precision Validation

**Figure 13:**
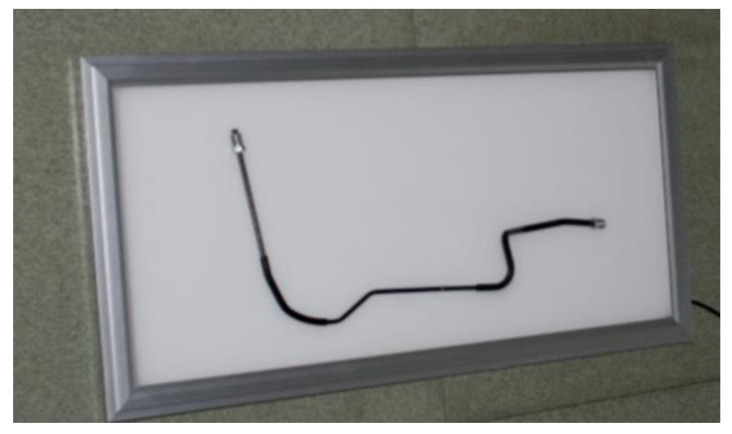
Precision validation experiment.

A tube is used to verify the effectiveness and accuracy of the proposed method. The parameters of the tube are measured by the laser scan method. Six cameras are calibrated by the photogrammetry method [25,26]. A LED plate light is used to make the line edge clear. The experiment is illustrated in Figure 13.

**Figure 14:**
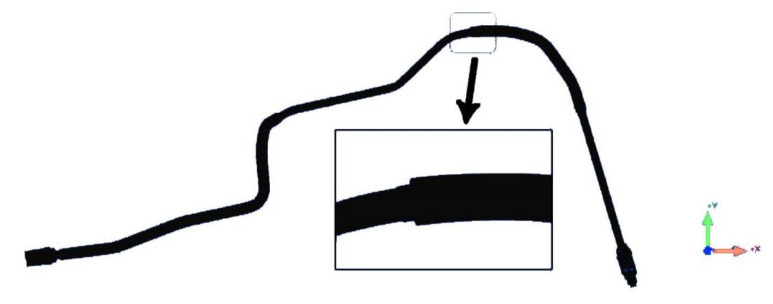
Tube reconstruction result.

Figure 14 shows the tube reconstruction result. According to the reconstruction result, the whole tube has been reconstructed. Different diameters are reconstructed well. The direction and dimensions of the connections at both ends of the tube were reconstructed successfully. Because of the reconstruction step threshold, there was a deviation in fitting a cylinder diameter where the diameter suddenly changes.

**Figure 15:**
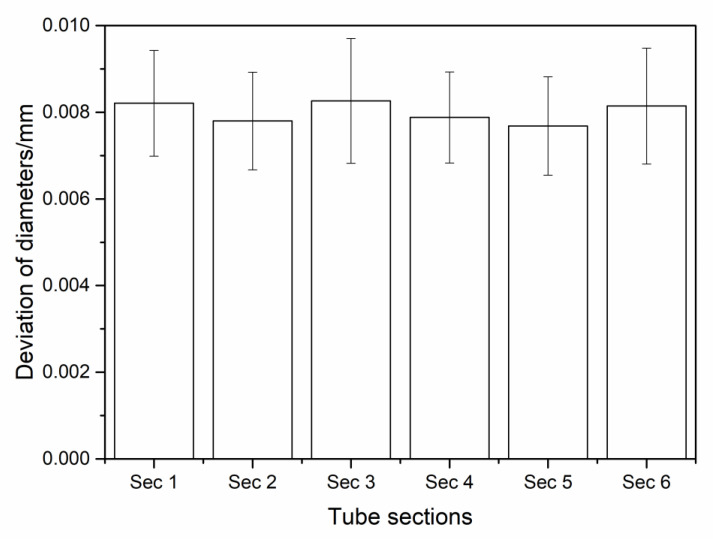
Deviation of diameters in the precision validation experiment.

According to the shape of the tube, divide the tube into six sections and seven bends. Each section of the tube had a similar diameter. The reconstructed diameters and bend angles of the proposed method were compared with the laser scan result. The comparison result is illustrated in Figure 15 and Table 1. The deviation of the diameters was less than 0.01 mm. The deviation of the bend angles was less than 0.015°. The comparison result shows that the proposed method has excellent precision for the measurement of the tube.

### 3.3. Complex Tube System Measurement

**Figure 16:**
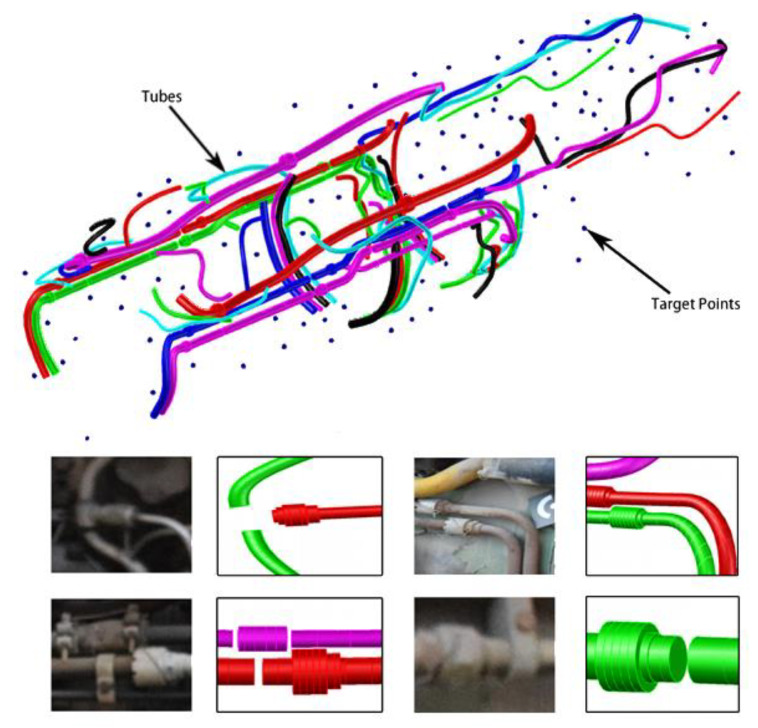
Complex tube system construction result.

An actual aero-engine was measured by the proposed method. The reconstruction result is illustrated in Figure 16. It can be seen that tube reconstruction was successful. Diameters of different tubes and distances between tubes were measured and compared with the contact measurement result to verify the accuracy and effectiveness of the proposed method.

**Figure 17:**
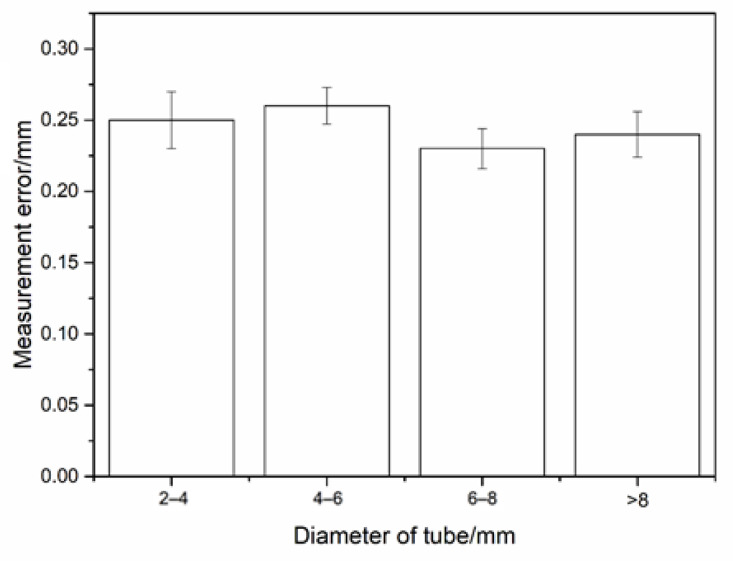
The measurement error of the complex tube system construction.

**Figure 18:**
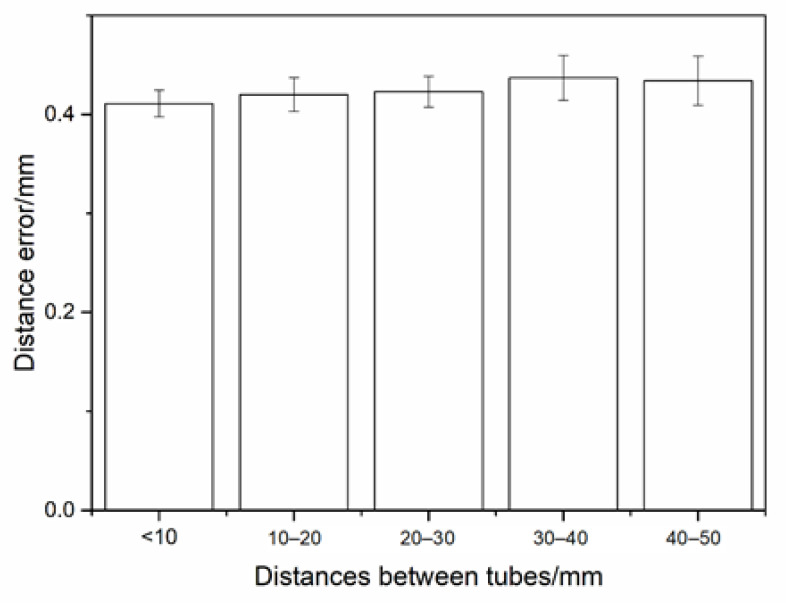
Distance error of the complex tube system construction.

The result of diameter comparison is illustrated in Figure 17. It can be seen that the reconstruction accuracy of tubes was not related to the diameter; the reconstruction error of the tube was less than 0.3 mm; the standard deviation was less than 0.02 mm. Distances between tubes were equally measured by plug gauge and standard blocks. Compared with the reconstruction result, the deviation was less than 0.5 mm, and the standard deviation was less than 0.1 mm. The error of distances result was slightly larger than the diameter result. As shown in Figure 18, the average distance error was less than 0.43 mm. The main reason is that the accurate minimum distance can be obtained by 3D reconstruction, while the measurement result of contact measurement is slightly larger than the actual minimum distance value.

## 4. Conclusions

A novel method of complex tube system reconstruction is proposed, which uses industrial photogrammetry to establish the spatial relationship between image acquirement stations. The complex tube system is reconstructed based on the space parameter constraint. The dimension and relationship of the complex tube system can be realized by this method. The experimental results show that the proposed method can be used to reconstruct the complex tube system effectively. It can effectively improve the detection efficiency and measurement accuracy of the complex tube system. For aerospace, industrial and chemical industries, this method can provide efficient measurement applications.

## Figures and Tables

**Table 1:** Deviation of angles in the precision validation experiment.

Bend	1	2	3	4	5	6	7
Deviation of bend angle/degree	0.014	−0.002	−0.013	0.007	0.013	−0.012	0.014

## Data Availability

Not applicable.
